# Gut bacteriome, mycobiome and virome alterations in rheumatoid arthritis

**DOI:** 10.3389/fendo.2022.1044673

**Published:** 2023-01-09

**Authors:** Sonali Dagar, Jagdeep Singh, Aastha Saini, Yashwant Kumar, Seema Chhabra, Ranjana Walker Minz, Lekha Rani

**Affiliations:** Department of Immunopathology, Postgraduate Institute of Medical Education and Research, Chandigarh, India

**Keywords:** gut microbiota, dysbiosis, rheumatoid arthritis, immune responses, adjuvant therapy

## Abstract

Rheumatoid arthritis (RA) is a chronic destructive autoimmune disease of the joints which causes significant pain, functional disability, and mortality. Although aberrant immune cell activation induced by the imbalance between T helper Th1/Th17 and Treg cells is implicated in the RA development, its etiopathogenesis remains unclear. The presence of mucosal inflammation and systemic IgA-isotype-autoantibodies (anti-citrullinated peptide antibodies and rheumatoid factor) in pre-clinical RA supports the mucosal origin hypothesis involving altered microbiota in disease development. The gut microbiota comprises diverse bacteria, fungal and viral components, which are critical in developing host immunity. Alterations in microbial abundance are known to exacerbate or attenuate immune responses in the gut microenvironment subsequently affecting the joints. Further, these changes can provide biomarkers for disease activity and outcome in RA. Most of the research till date has been focused on describing gut bacterial components in RA. Studies on gut mycobiome and virome components in RA are relatively new and burgeoning field. Given the paucity of mycobiome or virome specific studies in RA, this review, discusses the recent findings on alterations in gut bacterial, fungal, and viral components as well as their role in regulating the spectrum of immune-pathogenic events occurring in RA which might be explored in future as a potential therapeutic target. Further, we provide an overview on inter-kingdom interactions between bacteria, fungi, and viruses in RA. The current understanding on gut microbiota modulation for managing RA is also summarised.

## Introduction

Rheumatoid arthritis (RA) is an autoimmune disease characterized by synovial inflammation caused by leukocyte infiltration in the joints. It affects approximately 1% of the population worldwide (1). Although the pathogenesis of RA is not well understood, it involves complex interactions between immunological, genetic, and environmental factors (2, 3). Polymorphisms in genes such as HLA-DRB1, PTPN22, CTLA-4, and PADI4 are known to be associated with a genetic predisposition for RA pathogenesis. Environmental factors such as silica exposure, smoking, and infections also contribute to pathogenesis by inducing breakdown of immune tolerance to post-translationally modified proteins. These altered proteins are presented by dendritic cells (DCs) to T cells, which in turn activate B cells to undergo plasma cell differentiation leading to secretion of autoantibodies such as anti-citrullinated protein antibodies (ACPA) and rheumatoid factor (RF) (4, 5). These auto antibodies form immune complexes, which activate immune cells; these in turn recruit various other inflammatory cells to the joints contributing to localized destructive mechanisms. Moreover, DCs promote T-helper 17 (Th17) differentiation and inhibit regulatory T (Treg) cell differentiation, thus shifting the T cell balance toward inflammation. The activated T cells then stimulate effector cells such as macrophages, fibroblasts, osteoclasts, and chondrocytes, as well as their effector molecules, leading to cartilage and bone destruction (2–4).

Although the mechanisms underlying the RA pathogenesis remains elusive, altered gut microbiota composition, namely dysbiosis, influences the autoimmune responses and disease outcomes in RA (6–11). Most studies suggest that microbial colonization of gut begins during or after birth, whereas others suggest *in utero* acquisition probably through contact with placental microbiota, which originates from mother’s oral and gut microbiome (12–14). However, the evidence supporting *in utero* colonization are weak, further supporting its acquisition during and after birth (15). Gut microbiota is less diverse at the time of birth, however, their composition, abundance, diversity is significantly influenced by diet, the feeding mode (breast-fed or formula-fed) and during infant transition from milk to solid food within the first three years of life (16–18).

Among the different microbes in the gut microbiota, bacteria are the most prevalent. The gut is also colonized by other microorganisms, such as viruses, fungi, and archaea, which play pivotal roles in modulating the mucosal barrier and immune responses (19).

In this review, we discuss gut dysbiosis with an emphasis on the bacteriome, mycobiome, and virome in RA. Further, we explain how these three components of the gut microbiome interact to influence the disease pathophysiology. Further, we overview the mechanisms underlying the activation of innate and adaptive immune responses and link intestinal dysbiosis with the development and perpetuation of RA. Finally, we propose a few strategies on modulating the gut microbiota as potential treatment approaches in RA.

### Methods

Google and PubMed searches were performed using the terms ‘microbiota in RA,’ ‘gut-joint axis in RA,’ ‘microbiome,’ ‘bacteriome in RA,’ ‘gut fungi in RA/autoimmune diseases,’ ‘virome in RA/autoimmune diseases,’ ‘inter-kingdom interactions’, ‘diet,’ and ‘probiotics.’

## Gut microbial dysbiosis in RA

Accumulating evidence has shown the association of an altered gut microbiota with RA pathogenesis, which triggers arthritis based on genetic susceptibility and host-related factors (10, 20).

### Bacteriome in RA

Studies comparing the gut microbiota in patients with RA and in healthy controls have revealed enrichment of gram-positive bacteria and reduction of gram-negative bacteria. The enriched gram-positive bacteria predominantly comprise *Eggerthella lenta, Clostridium asparagiforme, Lachnospiraceae* bacterium, and *Gordonibacter pamelaeae*, which can be normalized after treatment (8). Among bacteria, *Prevotellaceae* is the major bacterial family associated with dysbiosis. Various studies observed the overexpansion of *Prevotella copri* in fecal samples from patients with RA (21–23). Further, a peptide derived from a 27 kDa protein from *Prevotella copri* (Pc-p27) was found to be presented on HLA-DR and was shown to stimulate a Th1 cell response in patients with new onset RA (21, 22). Along with *Prevotella*, other rare microorganisms have also been described in RA. Chen et al. demonstrated the abundance of *Collinsella* and *Eggerthella* in patients with RA, along with an overall reduction in intestinal diversity compared to that in the first-degree relatives (FDR) of RA patients and healthy controls (24). A study by David et al. showed increased abundance of *Clostridiaceae and Epsilonproteobacteria* in RA (25). Further, enrichment of *Lactobacillus salivarius* and depletion of *Haemophilus* species has been reported in the gut of Chinese patients with RA (8). Other recent studies indicated an increased abundance of *Klebsiella*, *Escherichia-shigella*, *Eisenbergiella*, *Lactobacillus, Streptococcus, Akkermansia* and *Flavobacterium* and depletion of *Bacteriodes fraglis*, *Faecalibacterium*, *Fusicatenibacter*, *Megamon*, *Bifidobacterium, Clostridium* and *Enterococcus* genera in RA (26–31). These studies suggest that bacteriome have a role in RA pathogenesis.

### Mycobiome in RA

Fungi represent only 0.1–1.0% of the intestinal microbiota and are referred as mycobiota (32). Although mycobiota compositions have not been very well examined, yeast has been shown to be most dominant species of the human mycobiome, particularly *Saccharomyces* (*S. cerevisiae*), *Malassezia* (*M. restricta*), and *Candida* (*C. albicans*) (33). At the phylum level, increased abundance of *Ascomycota* and decreased abundance of *Basidiomycota* has been reported in the synovial fluid of patients with RA (34). Among the predominant fungi, *C*. *albicans* is the most frequently detected, and has been associated with many autoimmune disorders (35–37). Further, fecal samples of Chinese patients with RA showed increased abundance of *Candida* and *Wallemia species* and decreased abundance of *Pholiota*, *Scedosporium*, and *Trichosporon* species (38). Furthermore, a lower frequency of fungal genera such as *Dendroclathra*, *Phacidium*, and *Septobasidium* was found in fecal samples from older patients than younger patients with RA (38). Previous *in vivo* studies have implicated mycotoxins in RA pathogenesis (39). In addition, Th17 cells mediate immune responses against fungi, also play critical role in the pathogenesis of RA, further suggests that the differences in the fungal microbiome might be related to inflammatory responses in RA (36, 37, 40).

### Virome in RA

Gut viruses, collectively called the virome, are a largely understudied component of the human gut microbiome (41). A case-control study reported that crAss-like phages are significantly reduced in RA and that the hosts of these crAss-like phages are *Bacteriodes vulgates* and *Firmicutes* (42). Yutin et al. reported *Prevotella copri* as a host for crAss-like phages (43). A recent study characterizing the fecal virome of the RA-FDR revealed increased *Bacteroidaceae*-infecting phages. On stratifying the participants based on serology, the samples from ACPA-positive FDRs were found to be more enriched in *Streptococcaceae*- and *Lachnospiraceae-*infecting phages than those from ACPA-negative FDRs (44). Ruochun et al. reported enrichment of *Lactococcus* phages in the dental plaques of treated patients with RA as compared to untreated RA. However, minor changes in the gut virome were observed along with a significantly decreased abundance of the family *Phycodnaviridae* in treated patients with RA (45). As the role of the bacteriome in autoimmunity development has been well-established (46), the changes of bacteriophage abundance in at-risk individuals suggests that they might be involved in RA pathogenesis, perhaps by modulating bacterial taxa or by directly interacting with the host immune system.

The alterations in bacteriome, mycobiome and virome components of gut are listed in **Table 1**.

**Table 1:** Alterations of gut bacteriome, mycobiome and virome components in RA.

Year	Increased Taxa	Decreased Taxa	Technology	Ref
Bacteriome				
2013	*Prevotella copri*	*Bacteroides*	16S sequencing and shotgun sequencing	(21)
2015	*L salivarius*	*Haemophilus spp*	Metagenomic shotgun sequencing	(8)
2016	*C. aerofaciens* and *E. lenta*	*Faecalibacterium*	16S sequencing & metabolomics	(24)
2019	Clostridiaceae, Epsilonproteobacteria	–	Metagenomic DNA Sequencing	(25)
2019	*Prevotella copri*	*Bacteroides* and *Bifidobacterium*	16S rRNA gene amplicon sequencing	(27)
2019	*Escherichia-Shigella & Bacteroides*	*Lactobacillus, Alloprevotella, Enterobacter *	Bacterial 16S rRNA sequencing	(28)
2019	*Bacteroides and Prevotella*	*Clostridium leptum*	qPCR	(29)
2021	*Lactobacillus, Streptococcus, and Akkermansia*	*Bacteroides, Faecalibacterium*	16S rDNA sequencing & LC-MS metabolomics	(30)
2022	*Klebsiella, Escherichia, Eisenbergiella and Flavobacterium*	*Fusicatenibacter, Megamonas and Enterococcus*	16S rDNA sequencing & LC-MS metabolomics	(31)
Mycobiome				
2019	*Aspergillus*, *Hypocreales*	*Malasseziales, Cladosporium*	Sequencing of the ITS2 region	(34)
2022	*Candida, Wallemia*	*Pholiota, Scedosporium*, and *Trichosporon*	ITS2 rRNA MiSeq sequencing	(38)
Virome				
2021	–	crAss-like phages	Shotgun sequencing	(42)
2021	Streptococcaceae Phages, *C. scindens*, and *A. oris* targeting phages	–	16S rRNA Amplicon Sequencing	(44)
2022	Myoviridae, Herelleviridae, Autographiviridae	Siphoviridae	Shotgun sequencing	(45)

## Crosstalk between the bacteriome, mycobiome and virome

The presence of intestinal bacteria limits the fungal colonization or viral invasion of the intestine and vice versa (47–49). Interactions between bacterial, fungal, and viral components in the gut have the potential to enhance pathogenesis. Although reports on inter-kingdom bacteria-fungi interactions in RA are limited, studies on patients with inflammatory bowel disease (IBD) indicate that bacterial and fungal interactions influence gut inflammation (50, 51). Hoarau et al. demonstrated positive inter-kingdom correlations between *E. coli*, *S. marcescens*, and *C. tropicalis* in patients with Crohn’s disease (CD) and that *C. tropicalis* cooperates with *E. coli*, and *S. marcescens* to form biofilms consisting of fungal hyphae and other species-specific interactions, which have pathogenic potential to damage host tissues (50). Sokol et al. also observed a positive correlation for inter-kingdom bacteria-fungi interactions of *Saccharomyces* and *Malassezia* with several bacterial taxa in patients with IBD (35). Further, a study using two model yeasts, *C. albicans* and *S. boulardi*, showed that the presence of *Enterobacteriaceae* is required for the effect of fungi on gut inflammation in a dextran-sulphate sodium induced colitis model (51). These findings suggest that bacteria-fungal interactions might influence the immune system in RA.

Analysis of the virus-bacteria interactions in the gut has reported a correlation between the abundance of crAss-like phages and *Bacteroides intestinalis* in autoimmune diseases, especially RA and SLE. Further, a positive correlation between two bacterial clades *i.e.*, *Faecalibacterium* spp. and *Faecalibacterium cf. prausnitzii* with *Podoviridae* abundance in patients with SLE was reported (42). *Faecalibacterium* is a bacterial genus which shows anti-inflammatory activity by producing short-chain fatty acids (52). Thus, the symbiotic relationship of *Faecalibacterium* and *Podoviridae* could be important to maintain immune homeostasis. Another study has reported that the bacteria-virus interaction network is disrupted in treated patients with RA compared to untreated patients and healthy controls (45). Moreover, phages can affect gut bacteria pathogenicity by altering their adhesion, invasion, colonization, and toxin production (53). Overall, these findings suggest that inter-kingdom interactions impact the host immune responses in RA.

## Mechanisms of immune activation in RA development and perpetuation of RA

Despite available evidence, the exact pathway by which dysbiosis specifically induces synovial inflammation remains poorly understood. The mechanisms by which bacterial dysbiosis favors RA initiation or progression include altered intestinal permeability, autoantigen modification, molecular mimicry, and immune/inflammatory system activation (54).

Alteration of intestinal permeability by the microbiota involves release of Zonulin, which decreases intestinal barrier function by displacement of the tight junction-forming proteins, zonula occludens 1 and occludins, from the junction complex, thereby increasing the penetrance of microbes or their products in the submucosa (55). Antigen presenting cells (APC) such as dendritic cells or macrophages respond to these microbes and their products and polarize T cells into Th1 and Th17 cells, which mainly produce IFN-γ and IL-17A, respectively (20). Group 3 innate lymphoid cells (ILC3) are pivotal in bridging the intestinal bacteriome and systemic immune responses (56). The penetrance of microbes and their products (ATP, free fatty acid receptor-2 agonists, aryl hydrocarbon ligands and retinoids) into the submucosa directly activates ILC3 and induces IL-22 secretion (57, 58). Other innate cells such as invariant natural killer T cells, mucosa-associated invariant T cells, macrophages, and monocytes also sense these bacteria and their products in the submucosa through various receptors such as Toll-like receptors (TLR), NOD-like receptors (NLR), c-type lectin receptors (CLR), and RIG-like receptors, thereby triggering the inflammatory cascade in the intestine and ultimately causing T cell activation and differentiation (20).

Another mechanism by which bacterial imbalance contributes to RA etiopathogenesis includes posttranslational modification of self-proteins such as hypercitrullination, which promotes ACPA production by inducing loss of T and B cell tolerance against citrullinated neoantigens (59–61). Some specific mucosa-associated bacteria have been implicated in promoting protein citrullination *via* activation of peptidyl-arginine deiminases after their release during cell damage (60). Autoantibodies promote RA pathogenesis by forming immune complexes with antigens, which activate innate immune cells by binding with Fc receptors as well as induce bone degradation by promoting osteoclastogenesis (62, 63).

In addition to autoantigen modification, mucosal and joint immune responses are considered to be linked by the molecular mimicry between certain bacterial antigens (epitopes from *Prevotella*) and autoantigens (N-acetylglucosamine-6-sulfatase). In the gut mucosa, *Prevotella-*derived epitopes are speculated to activate T cells, which then migrate to joints and cross-react with autoantigens to mount aberrant immune responses (64).

**Figure 1:**
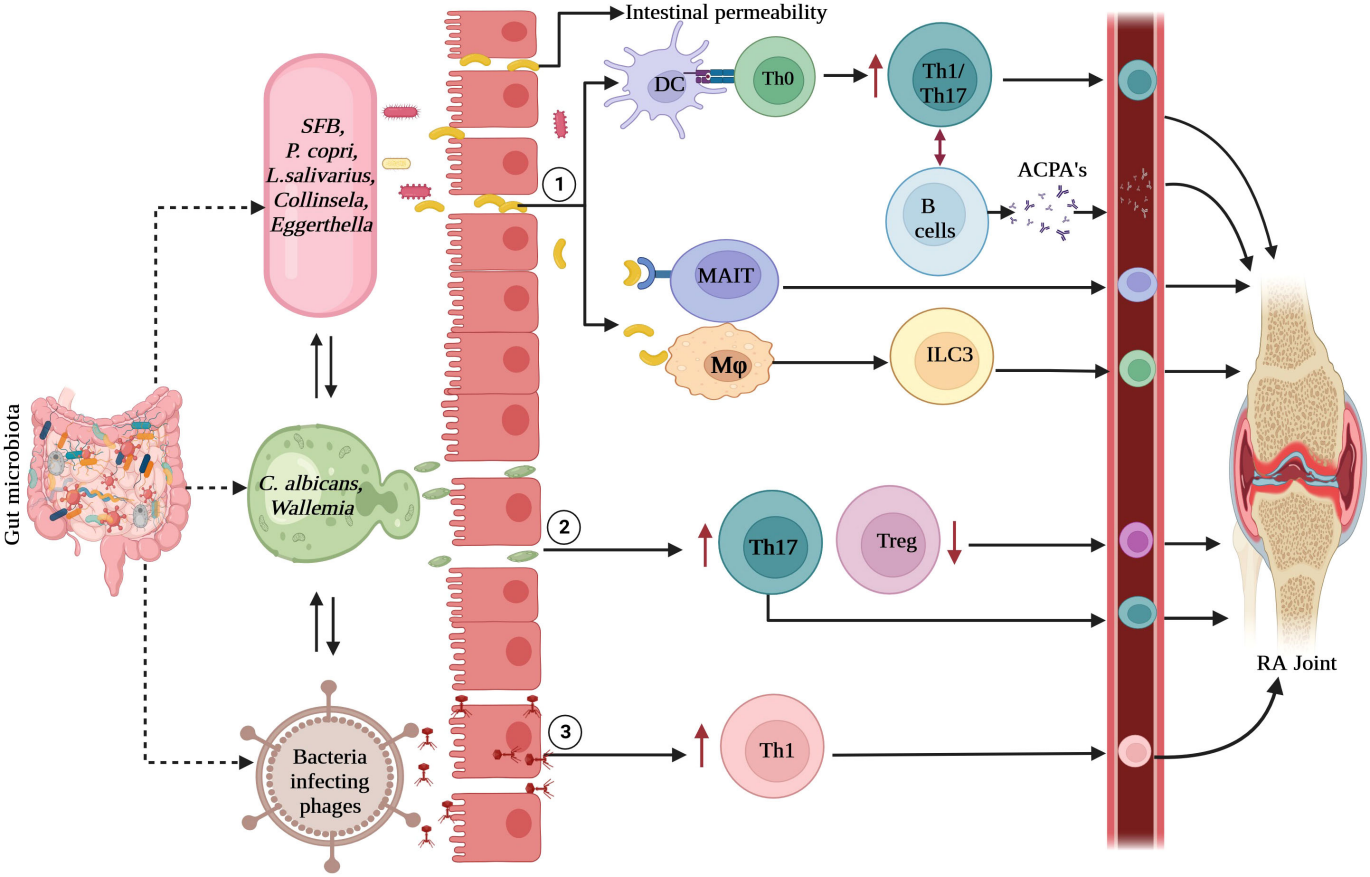
Interactions between gut microbiota and immune system: (1). Bacteria and its derived products activate gut epithelial and immune cells to enhance gut permeability which causes the migration of microbes into submucosa. These microbes are presented by dendritic cells to T cells which polarize into Th1 and Th17 cells. Gut colonization by SFB causes the induction of Th17 cells and *P. copri* and *L. salivarius* enhances the Th1 responses. These antigen-specific Th cells activate B cells to produce IgA-ACPA. Bacteria and its products directly activate macrophages and other innate immune cells such as ILC3 or MAIT cells which lead to gut inflammation locally and further activation of T cells. (2). The interactions between fungi mainly *C. albicans* and phagocytes leads to induction of increased Th17 responses and decreased Treg cells which contribute to local and systemic inflammation. (3). Viral dysbiosis causes induction of Th1 responses. Upon dysbiosis in RA, these innate and adaptive immune cells migrate to joints, exaggerating inflammation. RA, rheumatoid arthritis; Th, T-helper cells, ILC3, group 3 innate lymphoid cells; MAIT, mucosa associated invariant T cells; ACPA, anti-citrullinated peptide antibodies; SFB, segmented filamentous bacteria, *P.copri, Prevotella copri; L. salivarius, Lactobacillus salivarius; C.albicans, Candida albicans*.

Another mechanism in RA immunopathogenesis involves the induction of inflammatory immune responses. Certain bacteria, such as segmented filamentous bacteria (SFB), *Prevotella copri*, and *Lactobacillus* species increase Th17 and Th1 cell responses in the gut mucosa (22, 65–67). In addition to the bacteriome, minor components of gut microbiota such as fungi and viruses also contribute to immune response activation, which may lead to RA development. The interactions between the gut microbiota and immune system are depicted in **Figure 1**.

Under homeostatic conditions, immune responses to yeast and filamentous fungi involve their recognition by CLR such as Dectin-1, Dectin-2, and Mincle, expressed by gut mononuclear phagocytic (MNPs) cells such as CX3CR1^+^ MNPs, which induce fungi-specific Th17 responses that orchestrate protective immunity in intestinal tissues (68, 69). However, dysregulated Th17 responses contribute to local and systemic autoimmune conditions, such as IBD and RA (70, 71), therefore connecting the altered gut mycobiota with inflammatory diseases, including RA.

Although viruses represent only a minor component of the gut microbiome, they are one of the key regulators of host immune responses. Several studies have reported a correlation between virome fluctuations and RA; however, the mechanistic understanding regarding how these viruses contribute to RA pathogenesis remains limited (42, 44). The contribution of phages in exacerbating gut inflammation in IBD involves induction of Th1 immune responses *via* TLR9 activation on APCs in response to viral DNA (72). Overall, phages play a critical role in the pathogenesis of inflammatory diseases like IBD and RA, and could be considered a potential therapeutic target.

## Gut microbiota modulation as a potential treatment approach in RA

The advent and rapid evolution of disease modifying therapies has dramatically changed the concept of treatment in patients with RA. Additionally, these gut microbiome-modifying therapies raise the possibility that modulating the gut microbiome can be a promising therapeutic or adjunct strategy for treating RA. The microbiome can be manipulated by various interventions such as diet, vitamins, plant extracts, prebiotics, probiotics, and fecal microbiota transplantation (FMT) (73, 74).

Numerous meta-analyses indicate that oral supplementation of omega-3 poly unsaturated fatty acids significantly reduces the levels of inflammation-related markers (75, 76). Dietary fiber consumption helps prevent the gut microbiome from eroding the gut mucosal barrier, thereby reducing pathogen infection and enhancing the quality of life (77, 78). By managing metabolic imbalance and regulating the gut microbiota, an appropriated diet may progressively alter the physiological state as well as aberrant immune responses.

The potential of the gut microflora to influence immune responses has sparked significant interest in the use of probiotic microorganisms for both preventive and therapeutic purposes (79, 80). As defined by the World Health Organization, probiotics are “live bacteria” that impart a health benefit to the host (81, 82). *Lactobacillus* spp. and *Bifidobacterium* spp. are widely used probiotics. For instance, *L. casei* has been shown to reduce the induction and disease progression of adjuvant-induced arthritis by restoring microbiome dysbiosis in the gut (83). Further, the osteoprotective role of *Lactobacillus rhamnosus via* immunomodulation has been demonstrated in the management of bone-related diseases (81). *P. histicola* MCI 001, a therapeutic bacterial strain, has been shown to treat RA by increasing the synthesis of short chain fatty acids, which modulate the gut immune response (84).

Apart from bacteria, immune system-boosting fungal extracts, such as those from *Cordyceps militaris*, have the potential to be used as adjuvant therapy in treating immunological diseases (85). Further, consumption of *E. coli*-targeting phages leads to an increase in the butyrate-producing bacterial genus *Eubacterium* and a decrease in *Clostridium perfringens*, thereby indicating the potential of bacteriophages in treating immune-mediated diseases (86).

A number of organic substances or plant extracts, including clematis, berberine triterpenoid saponins, and *Paederia scandens* extract (PSE), have also been reported to influence the gut microbiota (73). PSE can effectively reduce the serum levels of TNF-α, IL-1β, IL-6, IL-7, and IL-23 in a mouse model of RA, suggesting that these plant extracts could be used as a potential treatment modality for RA (87).

Success of Fecal microbiota transplantation using healthy donor microbiota, is another method for treating gut dysbiosis (88, 89); however, the mechanisms by which FMT and FVT components exert beneficial effects need further investigation.

## Discussion

Gut microbiome plays a vital role in maintaining host immune homeostasis. Recent data from various studies indicate that dysbiosis occurs in pre-clinical phase of disease and influences the development of RA. Previous narrative reviews on gut microbiota are mainly focussed on bacteriome dysbiosis in RA. There is limited data on fungal and virome alterations in patients with RA. Further, inter-kingdom associations between bacteriome, fungal and viral components of gut have not been comprehensively evaluated till late. Therefore, this review discusses the available published literature on alterations in the gut biomes specially mycobiome and virome along with bacteriome in RA. Further, we are also narrating the role of gut microbial components in influencing immune system.

Studies showing the effect of dysbiosis on aberrant immune responses have opened new avenues of research and have already been exploited for novel treatment opportunities such as maintaining the gut barrier and inhibition of immune cell migration from the gut to joints. However, future studies are needed to define the causes of dysbiosis and to determine exactly how and when gut dysbiosis influences RA development.

As discussed in this review, most studies have demonstrated an association of the disease with altered microbial composition. However, a deeper understanding of this relationship and the mechanistic pathways influencing disease development is required for obtaining effective diagnostic, prognostic, and therapeutic targets. These findings provide guidance for manipulating the microbiome as a preventive, adjunctive, or therapeutic treatment strategy in RA. Over the past decade, several studies have shown that restoration of gut microbial homeostasis can be achieved *via* nutritional changes, administration of probiotics, and FMT. Therefore, clinical trials of these approaches are needed to determine the potential of gut microbiota modification as a component of therapeutic toolbox for RA.

## Author contributions

SD, JS and AS participated in the literature search and writing the review. YK, SC, RM provided valuable inputs in preparing and editing the manuscript. SD and AS created the illustrations. LR contributed to the conceptualization, design, writing, and editing of the manuscript. All authors contributed to the article and approved the submitted version.
